# Steroids in Lupus: Enemies or Allies

**DOI:** 10.3390/jcm12113639

**Published:** 2023-05-24

**Authors:** Eugenia Enríquez-Merayo, Maria J. Cuadrado

**Affiliations:** 1 Universitary Hospital 12 de Octubre, 28041 Madrid, Spain; eenriquez@unav.com; 2 School of Medicine, Universitary Clínica de Navarra, 28027 Madrid, Spain

**Keywords:** acrual damage, side effectis, dose, treatment duration

## Abstract

Glucocorticoids are the gold standard treatment for reducing immune activation and inflammation in a wide range of inflammatory and systemic autoimmune diseases. Glucocorticoids have potent and fast actions that quickly relieve some symptoms and lower mortality in some life-threatening conditions, but they also have side effects that limit the duration of treatment and the dose used. Systemic lupus erythematosus (SLE) is a systemic autoimmune disease characterized by the involvement of numerous organs and systems and the production of autoantibodies. Most current treatments include the use of corticosteroids and immunosuppressive medications. Glucocorticoids in SLE have been classically used not only to induce remission or treat an acute situation but also as maintenance therapy. During the last decades, new approaches to managing SLE have emerged, but corticosteroids continue to be part of all therapeutic regimes. There is more and more evidence about the side effects related to the use (or abuse) of steroids and their relationship with the accrual damage. In this manuscript, we try to make a critical review of the published literature about the benefit and side effects/damage that can be attributed to the use of glucocorticoids.

## 1. Introduction

Due to their powerful anti-inflammatory and immunosuppressive effects, glucocorticoids (GCs) are the standard treatment for many inflammatory and autoimmune disorders. GCs are frequently employed in a variety of medical specialties, including dermatology, rheumatology, nephrology, gastrointestinal, pneumology, and neurology. They serve as the gold standard treatment for reducing immune activation and inflammation in allotransplantation, allergic reactions, systemic autoimmune diseases, inflammatory bowel diseases, and various dermatological conditions.

GCs have potent and fast actions that quickly relieve some symptoms and lower mortality in some life-threatening conditions, but they also have side effects that limit the duration of treatment and the dose used.

Systemic lupus erythematosus (SLE) is an autoimmune condition driven by genetic and environmental factors that result in the malfunctioning of T cells, B cells, and dendritic cells, as well as the production of autoantibodies [1]. Most current treatments include the use of corticosteroids and immunosuppressive medications. These medications carry some toxicity concerns. Two monoclonal antibodies (Rituximab and Belimumab) used in the last decades offer a new approach.

The effects of corticosteroids are mediated through both genomic and non-genomic pathways. Knowing these mechanisms of action is clinically relevant due to their different side effects. The activation of one or the other pathway is dose and type of corticosteroid dependent.

Genomic pathways refer to long-term changes in gene expression that are caused by the binding of the drug to the intracellular cytoplasmic glucocorticoid receptor (cGR). When the drug binds to the receptor, it causes a conformational change, which activates the receptor. This, in turn, activates a series of intracellular signaling cascades, which ultimately lead to changes in gene expression. Upon binding to the receptor, corticosteroids induce the expression of genes that code the production of cytokines. The result is a decreased transcription of genes encoding inflammatory cytokines, a process known as transrepression, and an increased transcription of anti-inflammatory genes, known as transactivation. On the other hand, the non-genomic pathway involves changes that occur in a much shorter period. These pathways involve the direct binding of the drug to an intracellular receptor. Non-genomic effects are mediated through changes in cellular membranes, inactivation of the phospholipase A2 enzyme, and interaction with membrane glucocorticoid receptors (mGR). The final effect is decreased lymphocyte activity and proliferation [2,3].

Prednisone doses ranging from 7.5 mg to 30 mg (equivalent) per day activate genomic effects. High doses exceeding 30 to 50 mg per day gradually cause cGRs saturation [4]. Prednisone doses beyond 50 mg per day approach the limit of cGR saturation, with little additional anti-inflammatory benefit but an increased risk of side effects. Some of these side effects depend on the peak GC dose and length of high-dose exposure [5]. 

Most of the negative effects of glucocorticoids appear to be caused by transactivation, whereas the anti-inflammatory and immunosuppressive effects are thought to be caused by transrepression [6].

Glucocorticoids in SLE have been classically used not only to induce remission or treat an acute situation but also as maintenance therapy. During the last decades, new approaches to managing SLE have emerged, but corticosteroids continue to be part of all therapeutic regimes. There is more and more evidence about the side effects related to the use (or abuse) of steroids, and their relationship with the accrual damage in patients with SLE is confirmed in numerous studies. This is why the current practice aims to reduce the dose of steroids and the exposure time as much as possible to minimize damage.

## 2. Looking for Allies (Keep Your Friends Close)

The 2019 updated EULAR guidelines highlight the need to prevent organ damage, optimize pharmacological protocols to improve quality of life, and achieve long-term patient remission [7]. Treat to target in SLE aims to achieve remission or low disease activity without CS or with doses <7.5 mg/day [7,8]. Several studies, most of them in lupus nephritis (LN), and guidelines [8] support the combination of methylprednisolone (MP) pulses followed by doses of prednisone up to 30 mg/day. This regime is safer, more efficacious, and faster than the classical dose of 1 mg/kg/day.

The first study of SLE patients treated with high-dose intravenous MP (iv MP) pulses, published in 1976, described seven patients with lupus nephritis class IV. Renal function improved in 3 days by more than 50%, but improvement in lupus nephritis was not documented clearly [9]. In 1977, Ponticelli et al. presented 43 patients treated with iv MP pulses as the initial treatment of LN class IV [10], and 10 years later, the renal survival rate was more than 90% [11,12]. A double-blind clinical trial of 92 SLE nephritis patients compared MP pulses of 400 mg daily versus (vs) high-dose oral prednisolone and found more favorable and faster improvement in the iv MP group than in the oral group [13]. Several studies in the 1980s and 1990s demonstrated the usefulness of iv MP pulses not only in LN but in other severe manifestations of SLE, such as hemolytic anemia, neuropsychiatric lupus, and pulmonary hemorrhage, among others [12]. An NIH clinical trial performed in 1996 included 82 patients with proliferative lupus nephritis. They were randomized to receive a monthly dose of iv MP pulses alone or in combination with cyclophosphamide and a third arm with monthly cyclophosphamide (CYC) alone. The group with MP alone achieved lower remission rates than the combination groups, but in the long term, the MP alone and CYC combination groups had higher rates of renal response than the CYC alone group. It is uncertain whether monthly iv MP pulses produced additional benefits over daily high-dose prednisone in the combination group [14]. More recent studies support the initial data about iv MP pulses. Most of them are studies in LN and include dose regimens with iv MP pulses followed by medium oral glucocorticoids dose (<0.5 mg/kg/day). The Euro Lupus Nephritis Trial (ELNT) schedule included three 750 mg MP pulses, followed by 0.5 mg/kg/day of oral prednisone, slowly tapered to 10 mg/day by 6 months. This trial reported renal response rates (complete and partial) around 20 and 50% at 6 and 12 months, respectively, and long-term preservation of kidney function [14,15]. Most patients included did not clinically have severe kidney disease, and most of them were Caucasian, so the efficacy in other populations remains unclear [15,16]. Another clinical trial compared iv CYC vs. a combination of mycophenolate mofetil (MMF), calcineurin inhibitor, and GC. All patients received iv MP 500 mg/day pulses for three days followed by 0.6 mg/kg oral prednisone slowly tapered to 10 mg/day by week 16. The combination group achieved response rates of 86% vs. 63% in the CYC group, and 78% at 2 years of follow-up in both groups. This work has a 2-year follow-up period and includes only Chinese patients. It is well known that SLE has different clinical expressions and responses to treatment depending on ethnic groups. These results should be taken with caution when applied to other groups [17,18,19]. The “Rituxilup” scheme, consisting of rituximab and MP (500 mg on days 1 and 15), followed by maintenance treatment with mycophenolate mofetil without oral steroids, resulted in 72% of LN patients achieving complete remission. They were the first to show that treatment with oral steroids might be avoided in lupus nephritis [20]. The MYLUPUS trial compared the efficacy of medium-dose oral GC therapy (starting dose 0.5 mg/kg/day) to high-dose GC (starting dose 1 mg/kg/day). All patients received iv MP pulses (500 mg/day) for three days plus MMF. Complete and total response rates were similar at 6 months in both groups (CR:19 vs. 21% and PR: 67 vs. 56%). The authors conclude that LN can be treated with a lower steroid dose, with similar outcomes [21]. An uncontrolled single-center study to treat class III or IV LN includes the administration of iv MP pulses (3 days) with doses between 0.25–0.50 g and an extra pulse of 100 mg along with each cyclophosphamide bolus. The starting oral GC was <30 mg/day. In two reports, including 15 and 29 patients, response rates of 60% and 80%, and 86% and 87%, have been achieved at 6 and 12 months, respectively. The incidence of GC-related adverse effects was lower than in other historical cohorts [22,23]. A recent randomized, placebo-controlled, double-blind trial compared Voclosporin in combination with Mycophenolate and oral GC with a control group without Voclosporin for the treatment of LN (AURA-LV and AURORA). Patients received two 0.25–0.5 g methylprednisolone pulses, followed by a fixed 20–25 mg/d oral prednisone rapidly tapered to 5 mg by 12 weeks. This trial used lower oral GC doses and a faster tapering schedule than in other LN clinical trials. Significantly, more patients achieved a complete renal response (CRR) at one year in the Voclosporin group in the AURA-LR trial [24] than in the control group (43.7% vs. 23.3%). CRR at week 52 was achieved in significantly more patients in the Voclosporin group than in the placebo group (41% vs. 23%) in the AURORA trial [25].

The use of iv MP pulses contributes to reducing the starting oral GC doses and the time exposure to high GCs doses by allowing a faster tapering dose; however, what is the adequate dose of iv MP pulses that should be used? The classic tendency was to administer doses of 1 g per day, but there are several studies that suggest that the use of lower doses of iv MP is as effective as the classic dose, decreasing the adverse effects associated in the short and long term [26,27]. In 1987, 21 patients with severe refractory SLE were treated either with iv MP 100 mg/day or with 1000 mg/day for three days, without significant differences between the two groups. Despite including a small sample and the fact that the outcome criteria were not homogeneous, this study raised the possibility of using pulses with lower doses of iv MP [28]. Kong et al. reported in a retrospective study that 500 mg/day of MP (three days) was equally effective in controlling activity and had fewer serious infections compared to 1000 mg/day (3 days) and 1000 mg/day (5 days) doses [29]. A prospective study comparing 500 mg/day for three days with a historical cohort given higher doses did not find significant differences in patient outcomes [30]. Danza et al. [31] compared patients with several autoimmune diseases treated with iv MP pulses of 1500 mg, <1500 to 3000 mg, and >3000 mg (total dose over three days). They did not find differences between doses in patients achieving a complete response, partial response, or no response. No patients in the 1500 mg group suffered infections, vs. 9.1% in the other groups. Other studies demonstrated the efficacy of lower doses of iv MP pulse as seen above (ELNT, MYLUPUS, Rituxilup, “Lupus Cruces”, AURA, AURORA) [15,16,17,18,19,20,21,22,23,24,25]. Pulses of iv Prednisolone 125 mg, as a unique dose, have been successfully used in moderate flares to induce rapid remission and avoid higher oral doses for long periods [32]. It provides an interesting and novel vision of the management of mild flares. 

The maintenance dose of steroids in patients with SLE is also a subject of debate. Should we use maintenance doses of steroids? It seems that doses >7.5 mg/day of prednisone are related to more adverse effects. The current trend, according to EULAR clinical guidelines [7], is to reduce steroids as much as possible to minimize damage. However, many patients do not tolerate total steroid withdrawal, after long-term treatment, probably due to some degree of suppression of the hypothalamic-pituitary axis or due to relapse of SLE activity. Mathian et al. performed a monocentric, 12-month, superiority, open-label, randomized controlled trial to compare the efficacy of preventing flares of a maintenance dose of 5 mg/day versus total withdrawal of prednisone in SLE patients with clinically quiescent disease. The proportion of patients experiencing a flare was significantly lower in the maintenance group as compared with the withdrawal group (4 vs. 17). Maintenance of 5 mg prednisone was superior regarding the time to first flare. The authors concluded that long-term treatment with prednisone 5 mg/daily in SLE patients with inactive disease prevents relapse with no worsening of damage and no GC toxicity observed during the follow-up period compared with the withdrawal group. It is the only trial showing that low-dose prednisone can prevent flares, and although the follow-up time is only 12 months, it seems that it is safe and probably preferable in terms of accumulated damage to maintain low doses than the risk of new relapses [33]. Another study of the Hopkins Lupus Cohort also suggests that low doses of prednisone do not result in a substantially increased risk of irreversible organ damage [34]. In 2021, Ji et al. performed a systematic review and a meta-analysis to assess the risk of flare and damage accrual after discontinuation of low-dose glucocorticoids in SLE [35]. The meta-analysis describes a pooled incidence of flare of 24% and 13% for global and major flares, respectively. Pooled time to flare was 21.08 months. GC discontinuation showed an increased risk of flare compared with GC continuation, but the risk of major flares was not increased. GC withdrawal was associated with a borderline risk reduction in the SLICC/ACR damage index increase compared with GC continuation. The point is whether a low dose of steroids (below 7.5 mg/day) is acceptable to maintain clinical remission and to prevent flares or whether we should try to discontinue even low doses. The increased risk of flare would probably imply the administration of higher doses of steroids again [35]. In some patients, the endogenous production of cortisol is chronically impaired, so it is not possible to stop the steroid therapy [36]. Therefore, in selected patients in remission, on long-term steroid therapy, it might be acceptable to continue low-dose steroids as maintenance therapy.

In conclusion, glucocorticoid therapy is needed in SLE patients, mostly in two situations: severe or life-threatening conditions in which we need a rapid anti-inflammatory effect. In this case, an MP pulse of 500 mg per day is recommended. On the other hand, a low dose of GCs (equivalent to 5 mg/day of prednisone) may be necessary to maintain remission.

## 3. But Your Enemies Closer

Glucocorticoids have been shown to be useful in certain situations for SLE patients, but their use is associated with several adverse events and increased organ damage. The SLICC/ACR damage index (SDI) score is an important predictor of morbidity and mortality in SLE [37]. Clinical studies have shown that high doses (>30 mg/day) of glucocorticoids increase toxicity, including irreversible damage, whereas low doses or short-term pulse therapy have good safety profiles [22,27]. Within the first 5 years of diagnosis, at least half of SLE patients experience organic damage [38], primarily related to disease activity in the early stages and the use of glucocorticoids in later stages [39]. In a study of the Hopkins Lupus Cohort, a 1 mg/day increase in prior prednisone dose during follow-up was associated with a 2.8% increase in the risk of developing new organ damage. Higher doses of ≥7.5 mg/day versus <7.5 mg/day were significantly associated with an increased risk of cataracts (HR = 2.41), osteoporotic fractures (HR = 2.16), and cardiovascular damage (HR = 1.54) for individual organ systems [40]. The mean duration of follow-up was 6 years and included almost 2300 patients, which gives us important information about what happens in the first years of the disease. Longer follow-up time would be needed to evaluate the long-term involvement of steroids in organic damage. Another study from the same cohort estimated the effect of cumulative prednisone dose on damage accrual in SLE. The doses were divided into five levels (0, <180 mg/month, 180–360 mg/month, 360–540 mg/month, and >540 mg/month). The authors found that the risk of damage accrual increased by 1.16 with a cumulative dose of 180 mg per month (equivalent to 6 mg/day) and 2.51 for a cumulative dose >540 mg per month compared to patients without steroids. These data suggest that low doses of prednisone do not result in a substantially increased risk of irreversible organ damage, but a prednisone dose >7.5 mg significantly increases the risk of accrual damage [31]. The authors do not refer to patients’ compliance with the treatment.

The various complications associated with glucocorticoids depend on the dose and duration of exposure. Table 1 provides an overview of the adverse effects based on these factors. SLE patients are more prone to serious infections caused by high-dose intravenous MP used for severe disease activity [12]. Bashda et al. found that low albumin levels and concurrent use of cyclophosphamide increase the risk of infections in patients receiving MP pulses. The authors explain these results based on the binding of methylprednisolone to albumin alone, while prednisolone binds to both albumin and cortisol-binding globulin [41]. However, a systematic review concluded that the risk of infection with high doses of GCs appears to be independent of the concurrent use of immunosuppressive drugs [42]. 

SLE patients with kidney disease have an increased risk of severe infections [43]. Opportunistic and bacterial infections are more likely as the steroid dose increases from medium to high [44,45,46]. The most common infections in SLE are bacterial infections in the lungs, skin, and urinary tract [47,48,49]. As previously mentioned, studies have suggested that an MP bolus of 500 mg/day (for three days) followed by no more than 30 mg/day may lower the incidence of steroid-related infections in patients with LN.

Metabolic syndrome is a well-known adverse effect of long-term and high doses of GC therapy [50]. The risk of hypertension has been associated with the duration of GC exposure and with the daily dosage [51,52,53]. Insulin resistance increases in SLE patients receiving >7.5 mg/day of GC [54] in non-diabetic SLE patients is greater the more chronic the treatment with steroids and the risk of diabetes is [51,55,56]. Weight gain is frequently reported in GC-treated patients and is associated with >5 mg/day of prednisone [57,58].

The risk of cardiovascular disease is higher in SLE patients than in the normal population. It is particularly high in patients with LN and in GC therapy >20 mg/day of prednisone. The use of medium to high-dose GCs has been associated with a high risk of cardiovascular events, subclinical atherosclerosis, and the severity of coronary calcifications [59].

Musculoskeletal GC-related adverse events are varied. Avascular necrosis has been described in association with steroid pulses, the peak initial GC dose, and the high-accumulated dose in the first months of treatment [60]. It has also been described more frequently in patients with LN [60,61]. Osteoporosis has been associated with higher GC doses, long-term therapy, and cumulative doses [57]. One study of the Hopkins Lupus Cohort estimated an increased risk of osteoporotic fracture of 4.2% for each mg per day of prednisone [40]. A systematic review to investigate the rate of adverse events of medium vs. high doses of GC in SLE published in 2017 pooled data from 8 randomized control trials and found a rate of 9/100 patients/year for hyperglycemia/diabetes, 25/100 patients/year for infections, and 12/100 patients/year for avascular necrosis of the hip. There were no differences in adverse events between patients receiving high or medium doses of GC [62]. This systematic review also supports the use of low doses of steroids to decrease the risk of osteoporosis.

The use of GCs in SLE patients is associated with damage accrual and with increased mortality, so we need to minimize the use of this therapy.

We must look for strategies to reduce the cumulative dose of steroids. Among them would be the administration of low-dose boluses (125–500 mg) in severe situations or even in moderate flares. Therapy with hydroxychloroquine is universally recommended [7] since it has been shown to have a protective effect on cumulative damage, infections, and mortality [63,64,65]. With or without a low GCs dose helps to maintain remission [66,67,68]. The introduction of immunosuppressive and/or biologics drugs also helps to decrease and even avoid treatment with GC.

## 4. Conclusions

Corticosteroids save lives in serious/life-threatening situations, so they will continue, at least in the coming years, to play a main role in the management of SLE patients. However, the actual tendency is to treat with low doses of steroids “as low as possible”. There is enough evidence demonstrating that chronic damage is associated with steroid treatment increasing morbidity and mortality. It also has an important impact on quality of life. Therefore, the therapeutic strategies should aim to discontinue steroid treatment as soon as possible or use a maintenance dose lower than 5–7.5 mg/day.

## Figures and Tables

**Table 1:** Side effects based on dose and treatment duration.

Low Doses (<5–7.5)		High Doses		
Early (<6 Months)	Long-Term (>6 Months)	Immediate Pulses	Early (<6 Months)	Long-Term (>6 Months)
Osteoporosis Hyperglycemia Cushing syndrome Hypertension Glaucoma Psychiatric disease Sleep disorders Dermatological	Cataracts Psychiatric disorders Osteoporosis Infections Cardiovascular disease Dermatological	Acute vascular events Hyperglycemia Hypertension, Avascular necrosis Psychosis	Cardiovascular and cerebrovascular events Avascular necrosis Myopathy Mood disorders Psychiatric disorders Insulin resistance Dyslipidemia Glaucoma Osteoporosis Dermatological	Infections Cushingoid features Insulin resistance Osteoporosis Cataracts Glaucoma Dermatological Infections Dyslipidemia Hypertension

## Data Availability

Not applicable.
